# Supplementary material for: Atmospheric wind energization of ocean weather
Source: Nat Commun. 2025 Jan 30;16:1172. doi: 10.1038/s41467-025-56310-1 (PMC11782504; doi:10.1038/s41467-025-56310-1)
Supplement: Supplementary file 1 — Supplementary Information [file 41467_2025_56310_MOESM1_ESM.pdf]

Atmospheric Wind Energization of Ocean Weather

Shikhar Rai[1,2], J. Thomas Farrar[2], and Hussein Aluie[1,3,4] *

[1]Department of Mechanical Engineering, University of Rochester, Rochester, New York, USA
[2]Department of Physical Oceanography, Woods Hole Oceanographic Institution, Woods Hole, Massachusetts, USA
[3]Department of Mathematics, University of Rochester, Rochester, New York, USA
[4]Laboratory for Laser Energetics, University of Rochester, Rochester, New York, USA
*Corresponding author: Hussein Aluie, hussein@rochester.edu

Supplementary Information

Supplementary Text

Strain in Divergence-free Flows

An occasional misconception is that strain is solely due to the potential flow component, $\nabla\phi$, which accounts for convergence/divergence. In fact, strain is also an essential constituent of divergence-free (or solenoidal) flows. Consider the Helmholtz decomposition of a 2-dimensional velocity field,

$$\mathbf{u} = \underbrace{\nabla\phi}_{\substack{\text{accounts for}\\ \text{strain only,}\\ \text{incl. divergence}}} + \underbrace{\nabla\times(\psi\,\hat{\mathbf{n}})}_{\substack{\text{divergence-free,}\\ \text{accounts for both}\\ \text{vorticity and strain}}} , \tag{S-1}$$

where ψ is a streamfunction and $\hat{\mathbf{n}}$ is the unit vector normal to the flow plane. The streamfunction ψ can be proportional to sea-surface height in a geostrophic flow, for example, such as that from satellite altimetry we analyze in the paper. To see how the divergence-free component $\nabla\times(\psi\,\hat{\mathbf{n}})$ accounts for strain, consider its symmetric gradient,

$$\frac{1}{2}\nabla[\nabla\times(\psi\,\hat{\mathbf{n}})] + \frac{1}{2}\nabla[\nabla\times(\psi\,\hat{\mathbf{n}})]^{\text{tr}} = \begin{pmatrix} \partial_x\partial_y\psi & \frac{1}{2}(\partial_y\partial_y\psi - \partial_x\partial_x\psi) \\ \frac{1}{2}(\partial_y\partial_y\psi - \partial_x\partial_x\psi) & -\partial_x\partial_y\psi \end{pmatrix}. \tag{S-2}$$

The strain in eq. (S-2) is generally non-zero almost everywhere in the flow and is dominant in saddle regions between vortices [1]. The strain tensor in eq. (S-2) is traceless, reflecting the divergence-free nature of the velocity component $\nabla\times(\psi\,\hat{\mathbf{n}})$ in eq. (S-1).

Wind Work on Vorticity and Strain

Okubo-Weiss Analysis

A common method to decompose flow into strain and vorticity is based on the Okubo-Weiss parameter [1, 2],

$$O = s_n^2 + s_\gamma^2 - \omega^2 , \tag{S-3}$$

where $s_n = \partial_x u_x - \partial_y u_y$ is proportional to the diagonal components of the deviatoric (traceless) strain rate tensor, $s_\gamma = \partial_x u_y + \partial_y u_x$ is proportional to the shear strain rate components, and relative vorticity $\omega = \partial_x u_y - \partial_y u_x$ is proportional to the components of the rotation rate tensor. The Okubo-Weiss parameter can be evaluated at any location $\mathbf{x}$. If $O > 0$, the location is classified as strain-dominated. If $O < 0$, the location is classified as vorticity-dominated. Therefore, the classification is binary and does not account for the co-existence of strain and vorticity at every location.

Using the Okubo-Weiss parameter, the mesoscale wind work in eq. (1) in the main text can be partitioned into wind work on vorticity-dominated regions, EP^{vort}, and wind work on strain-dominated regions, EP^{strn}. Maps of EP^{vort} and EP^{strn} are shown in Fig. S2 using satellite data and in Fig. S4 using CESM model data. When compared to Fig. 2 in the main text and Fig. S3 using our multiscale approach, we can see that on one hand, Figs. S2,S4 are qualitatively consistent, which offers support to our approach. On the other hand, Figs. S2,S4 highlight shortcomings of traditional approaches based

on pattern detection. We can see that maps in Figs. S2,S4 are washed out (zero wind work) due to the binary nature of the Okubo-Weiss criterion described above. This is because if, for example, a region is classified as strain-dominated within the Okubo-Weiss approach, wind work on vorticity is necessarily zero even though significant vorticity can be present in that region (albeit subdominant to strain). This can lead to severe errors in the bulk energy transfer estimates, which are especially pronounced in model data (Fig. S3A versus Fig. S4A) where damping estimated from Okubo-Weiss is a mere 15% of the damping revealed by our approach. Another important shortcoming of pattern detection approaches, such as Okubo-Weiss, is the inherent difficulty of deriving budgets governing coherent structures. This difficulty stems from the fact that the mask used to partition the flow into different structures is itself an implicit (and complex) function of the flow.

Similar to our approach in the main text, vorticity-dominated regions can be further partitioned into those with positive and negative vorticity. Wind work on these respective regions is then $EP^{vort}_{\zeta>0}$ and $EP^{vort}_{\zeta<0}$. Strain-dominated regions can also be partitioned into those with positive and negative strain based on the angle θ of the diverging arm of the local strain as done in the main text (see Fig. 2F). Maps of $EP^{vort}_{\zeta>0}$, $EP^{vort}_{\zeta<0}$, $EP^{strn}_{\theta>0}$ and $EP^{strn}_{\theta<0}$ based on Okubo-Weiss are shown in Figs. S9,S8 using satellite and model datasets, respectively.

When compared to their corresponding Figs. 3 (in the main text),S7 using our multiscale approach, Figs. S9,S8 highlight how poor Okubo-Weiss is at detecting the inherent asymmetry of energy transfer. Panels in Figs. S9,S8 are washed out due to the binary nature of the Okubo-Weiss. This is clear from the energization time-series in panels [G], where negative vorticity in the NH (orange) and positive vorticity in the SH (blue) oscillate around zero in contrast to the corresponding plots in Figs. 3 (in the main text),S7 which are clearly positive and indicate energization of anticyclonic flow by winds in the subtropics. Also missing from panels [G-H] is the regular seasonal signal we see clearly in Figs. 3 (in the main text),S7 along with a significantly weaker correlation coefficients r. This is because a vorticity-dominated region as detected by Okubo-Weiss can also have significant contributions from strain of either sign and vice versa, which contaminate the time-series.

Results from CESM

Our results from satellite data are reinforced by data from high resolution fully coupled Community Earth System Model (CESM) simulation [3]. The simulation is a 100-year run of fully coupled simulation with the atmospheric component modeled on a $\approx 0.25^\circ$ grid and the ocean component on a $\approx 0.1^\circ$ grid. The atmospheric component is the Community Atmosphere Model (CAM5) version 5 with Spectral Element (SE) dynamical core, and the ocean component is the Parallel Ocean Program (POP2). Further simulation details can found in [3]. Daily averaged wind stress and sea surface height (SSH) output variables from simulation years 50 to 56 have been used in this work. Geostrophic ocean surface currents were calculated using SSH outside $\pm 3^\circ$.

Supplementary Figures

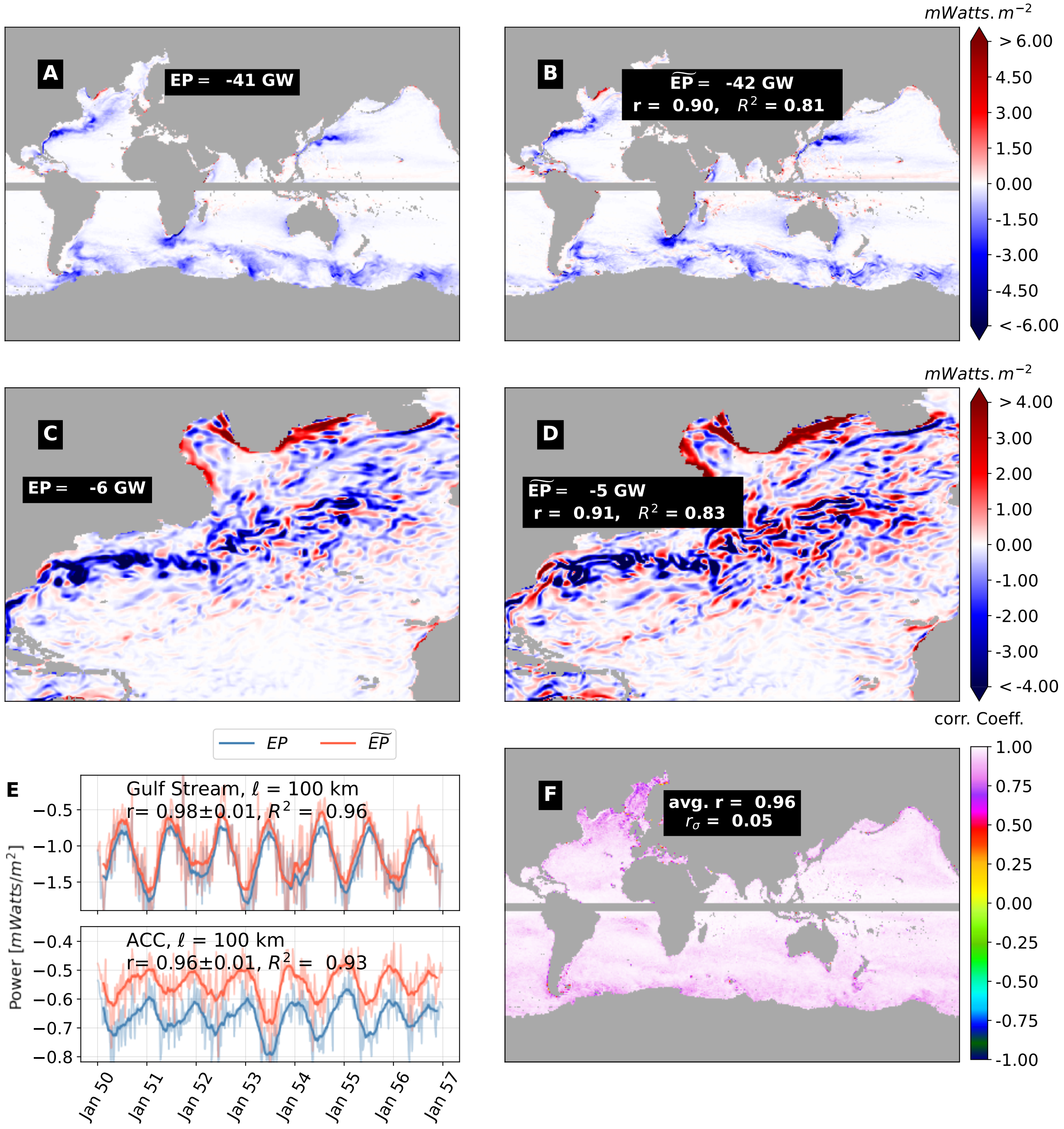

Fig. S1: CESM dataset: $\widetilde{EP}$ is an excellent proxy for EP. Same as in Fig. 1 in the Main text but using data from the high-resolution coupled ocean-atmosphere CESM simulation. [A] and [B] compare EP and $\widetilde{EP}$ (averaged from years 50 to 56) at $\ell = 100$ km. [C] and [D] compare EP and $\widetilde{EP}$ on a single day (Feb. 25, year 50) in the Gulf Stream region. These panels demonstrate that EP in [A],[C] can be accurately approximated by $\widetilde{EP}$ in [B],[D], which display a high Pearson correlation coefficient $r \approx 0.9$. The coefficient of determination, $R^2 \approx 0.9$ in [B], [D], and [E] shows the fraction of the variance of EP that can be described by a linear relationship between EP and $\widetilde{EP}$. [A]-[D] also display the area-integrated values of EP or $\widetilde{EP}$ (in GW). [E] shows time series of EP and $\widetilde{EP}$ in the Gulf Stream and in the ACC, which again exhibit high correlation (r = 0.97 and 0.98) and demonstrates that $\widetilde{EP}$ captures the seasonality of EP reported recently [4, 5]. Regions are defined as in [4]. [F] is a global map of the temporal correlation between EP and $\widetilde{EP}$ at every location, and shows an average correlation coefficient $r = 0.96$ ($r_\sigma = 0.05$ is its standard deviation), which reinforces our treatment of $\widetilde{EP}$ as an accurate proxy for EP. The $\pm 3^\circ$ gray strip at the equator is masked out since we don't calculate the geostrophic ocean velocity there.

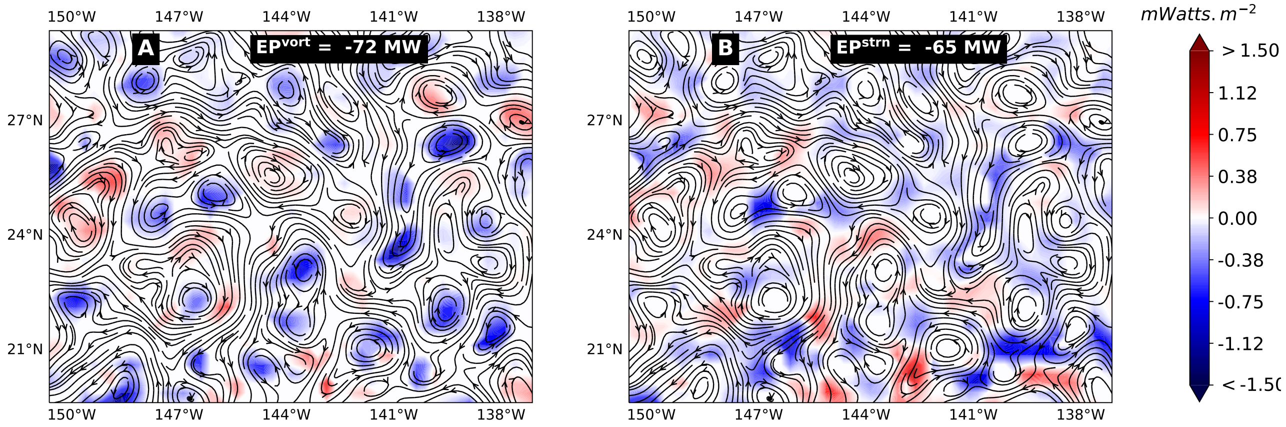

Fig. S2: Using Okubo-Weiss to decompose energy transfer to vorticity and strain. Similar to Fig. 2A-B (in the Main Text), but using the Okubo-Weiss criterion [1] to partition the flow into [A] vorticity and [B] strain regions using a mask function. After calculating EP_ℓ from eq. (1) (in the main text), the mask projects EP_ℓ onto [A] vorticity-dominated and [B] strain-dominated regions. On one hand, this figure provides support to our approach by showing results that are qualitatively consistent with Fig. 2A-B. However, the figure here also highlights shortcomings of traditional approaches based on eddy detection. First, the Okubo-Weiss criterion is a binary designation of a geographic location as either strain-dominated or vorticity-dominated even though strain and vorticity are often collocated. This is manifested as washed out white regions (zero wind work) in either [A] or [B], wherever there is a colored (non-zero wind work) in the other panel at the same location. This can lead to significant errors in bulk energy transfer estimates, which are especially pronounced in model data (Fig. S3A vs Fig. S4A) where damping estimated from Okubo-Weiss is a mere 15% of the damping revealed by our approach. Second, and more importantly, it is intractably difficult to derive budgets governing coherent structures that are obtained using traditional eddy detection methods such as Okubo-Weiss. This is because the mask used to partition the flow into different structures is itself an implicit (and complex) function of the flow.

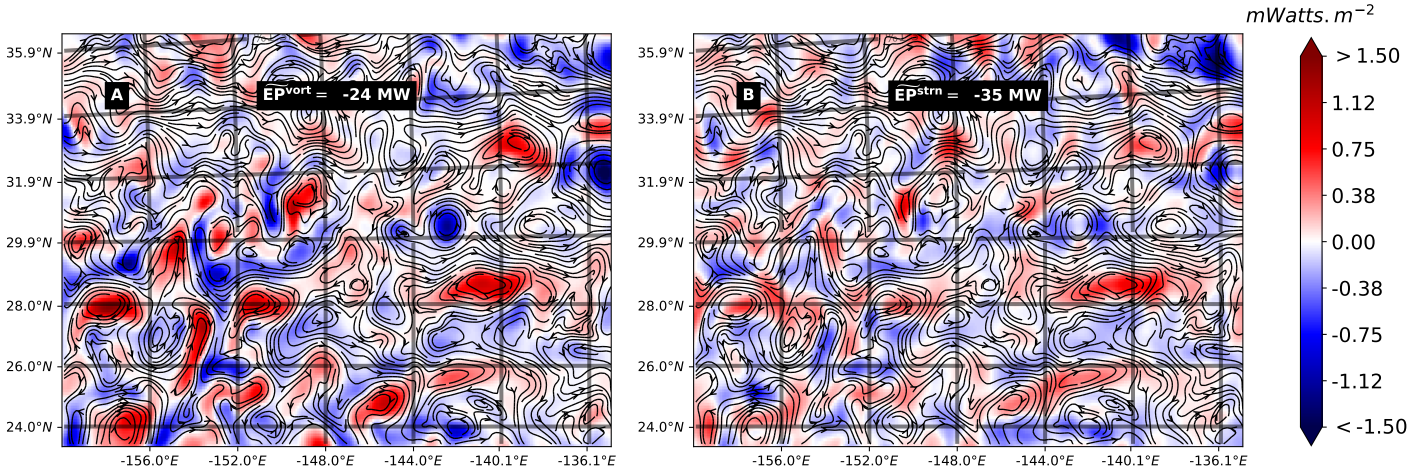

Fig. S3: CESM dataset: Disentangling Ocean Weather Energization by Winds. Same as in Fig. 2A-B in the main text, but using data from the high-resolution coupled ocean-atmosphere CESM simulation. Energy transfer (in Watts) from atmospheric winds into ocean [A] vorticity, $\widetilde{EP}^{vort}$ and [B] strain, $\widetilde{EP}^{strn}$, can be analyzed using our theory and are shown here in a region in the north Pacific on Feb. 25, simulation year 50. Streamlines in [A] and [B] are identical and visualize the ocean surface currents. While vortical and straining motions always co-exist at every location, some regions can be dominated by one or the other. Demonstrating our approach, [A] shows that wind work on vorticity is most pronounced inside ocean eddies (closed streamline contours), whereas [B] shows that wind work on strain has comparable magnitude but dominates in saddle-like regions outside eddies. Ocean-induced WSGs alone always damp ocean currents and do not explain the positive wind forcing (red) in [A] and [B]. This is explained by inherent wind gradients, which naturally arise in our theory, with an important component being due to the prevailing trade winds and westerlies sketched in Fig. 2F in the main text. These lead to asymmetric energization of ocean weather based on the polarity of vortical (anti/cyclonic) and straining ($\theta > 0/\theta < 0$) flows. Straight green lines are latitude and longitude lines from the tripolar CESM grid.

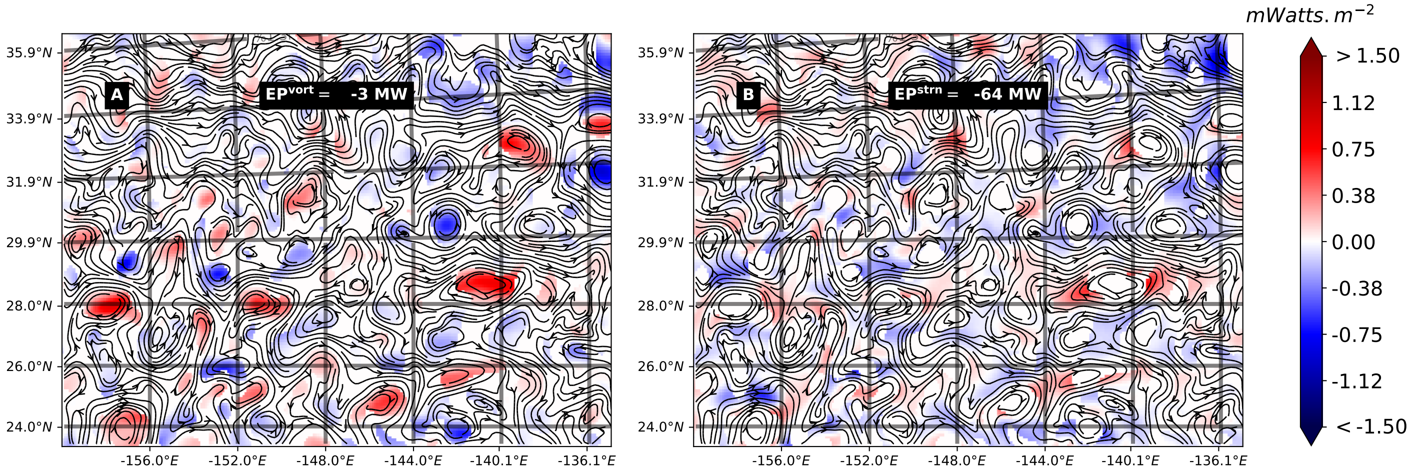

Fig. S4: CESM dataset: Using Okubo-Weiss to decompose energy transfer to vorticity and strain.. Similar to Fig. S3, but using the Okubo-Weiss criterion to partition the flow into [A] vorticity and [B] strain regions using a mask function. After calculating EP_ℓ from eq. (1) (in the main text), the mask projects EP_ℓ onto [A] vorticity-dominated and [B] strain-dominated regions. On one hand, this figure provides support to our approach by showing results that are qualitatively consistent with Fig. 2A-B (in the main text). However, as we found in Fig. S2 from the satellite dataset, the panels here are washed out due to the binary nature of the Okubo-Weiss criterion and highlights shortcomings of traditional approaches based on eddy detection. In [A], damping of vorticity is severely underestimated (-3 MW); it is only 15% of the energy transfer revealed by our approach in Fig. S3A (-20 MW). On the other hand, energization of strain in [B] is significantly underestimated compared to Fig. S3B leading to a bulk strain damping (-64 MW) that is more than twice the damping in (-30 MW). Most importantly, it is intractably difficult to derive budgets governing coherent structures that are obtained using traditional eddy detection methods such as Okubo-Weiss. This is because the mask used to partition the flow into different structures is itself an implicit (and complex) function of the flow.

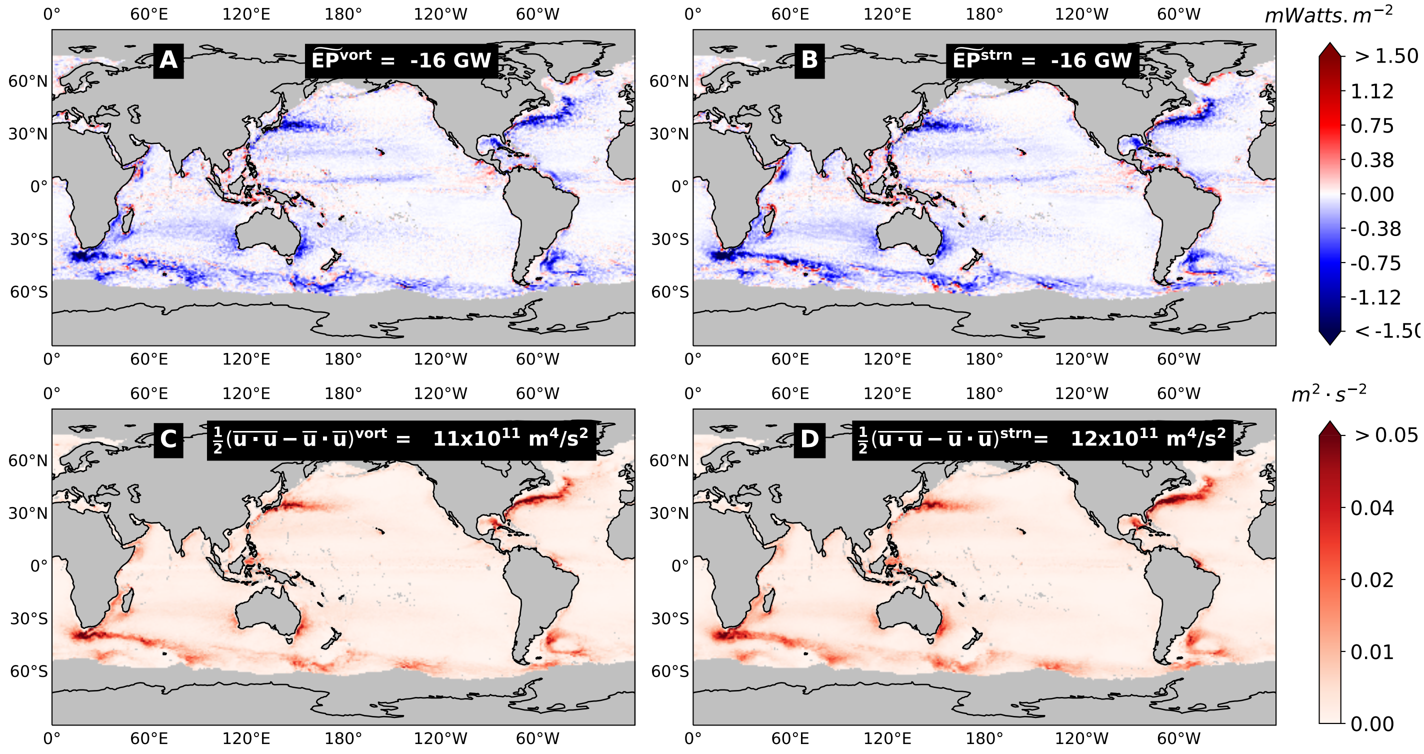

Fig. S5: Wind damping of mesoscale strain and vorticity. [A] and [B] decompose $\widetilde{EP}$ in Fig. 1B (in the main text) into wind work on mesoscale [A] vorticity and [B] strain. Panels show the time average (Oct. 1999 to Dec. 2006) using satellite data as in Fig. 1 (in the main text). [C] and [D] show mesoscale kinetic energy at scales $\ell < 100$ km for vorticity-dominated regions and strain-dominated regions. The vorticity and strain dominated regions are masked using Okubo-Weiss parameter. This figure shows that, on average, wind damps mesoscale strain and vorticity equally and that it is most pronounced in regions with high mesoscale kinetic energy.

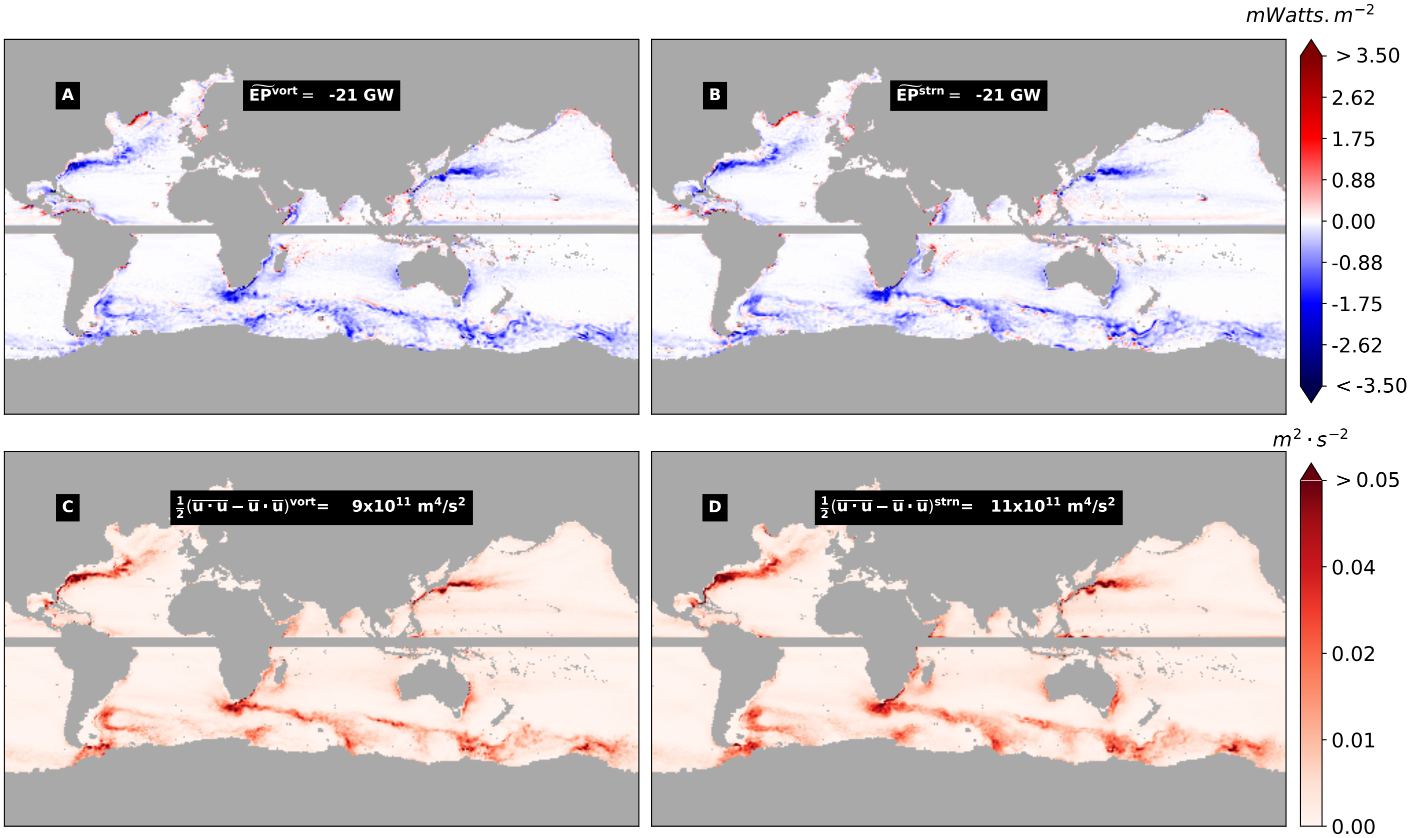

Fig. S6: CESM dataset: Wind damping of mesoscale strain and vorticity.. Similar to Fig. S5 but using CESM data. [A] and [B] decompose $\widetilde{EP}$ in Fig. S1B into wind work on mesoscale [A] vorticity and [B] strain. Panels show the time average (year 50 to 56) as in Fig. S1. [C] and [D] show mesoscale kinetic energy at scales $\ell < 100$ km for vorticity-dominated regions and strain-dominated regions. The vorticity and strain dominated regions are masked using Okubo-Weiss parameter. Similar to Fig.S5, this figure shows that, on average, wind damps mesoscale strain and vorticity equally and that it is most pronounced in regions with high mesoscale kinetic energy.

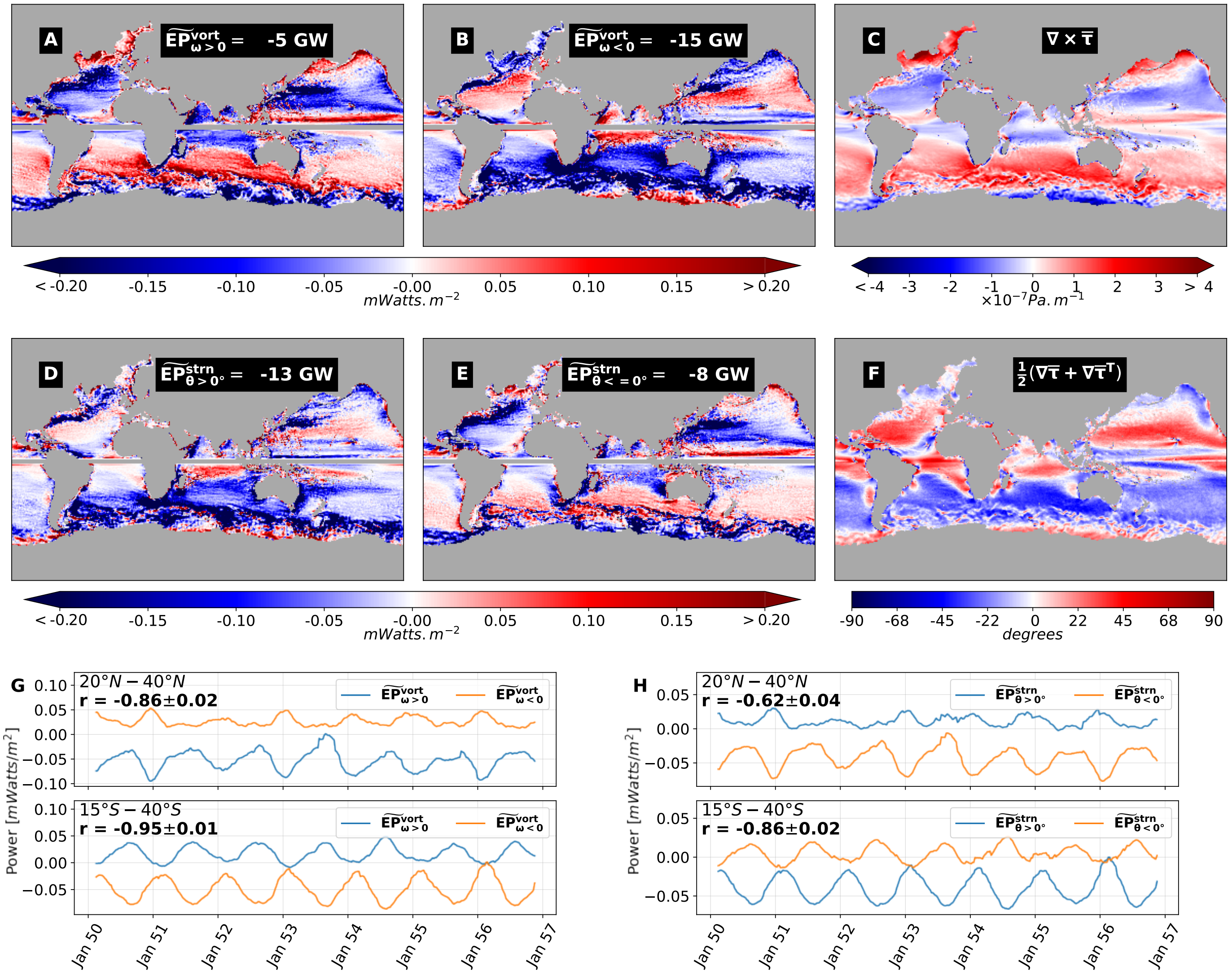

Fig. S7: CESM dataset: Unravelling Inherent Asymmetry of Energy Transfer from Winds to Ocean Weather. Same as in Fig. 3 in the main text, but using data from the high-resolution coupled ocean-atmosphere CESM simulation. Top/Bottom row shows the inherent asymmtery in wind energization of vortical/straining ocean mesoscale flows. [A]/[B] is wind work on flows with positive/negative (anti/clockwise) vorticity. In the subtropics ($[15^\circ - 45^\circ]$), cyclonic/anti-cyclonic vortices are damped/energized (blue/red) by winds and the reverse occurs in sub-polar regions. While eddy-damping dominates on a global average (Fig. 1A-B) (in the main text), anticyclonic vortical flows are in fact energized due to inherent wind stress gradients (WSGs) in most of the subtropical oceans, except in strong current regions where the eddies are sufficiently strong such that induced WSGs, which always oppose ocean currents, dominate. [C] is a map of the time-mean wind stress curl component of inherent WSGs acting on the ocean's mesoscales. Comparing [A-B] to [C] demonstrates the prevailing winds' imprint (see Fig. 2F in the main text) on the ocean's mesoscale vortical flow. [D-E] show wind energization of straining ocean flows with a positive/negative polarity ([D]/[E]) based on the angle θ of the local strain's diverging arm (Fig. 2F in the main text). [F] is the time-mean angle of the straining WSG, which again demonstrates the prevailing winds' imprint (see Fig. 2F in the main text) on the ocean's mesoscale straining flow. [G] is a (13 weeks running mean) time series of energization of flows with positive/negative vorticity (blue/orange) at latitudes $20^\circ N - 40^\circ N$ and $15^\circ S - 40^\circ S$, excluding strong current regions analyzed in [4] where damping by induced WSGs dominates (see Methods). There is clear seasonality in [G] with the vortical flow's energization/damping peaking during the local winter. [H] is similar to [G] but for the straining mesoscale ocean flow, which shows much the same seasonality. Coarse-graining in [A-H] is at scale $\ell = 100$ km. The $\pm 3^\circ$ gray strip at equator is masked out since we don't calculate the geostrophic ocean velocity there.

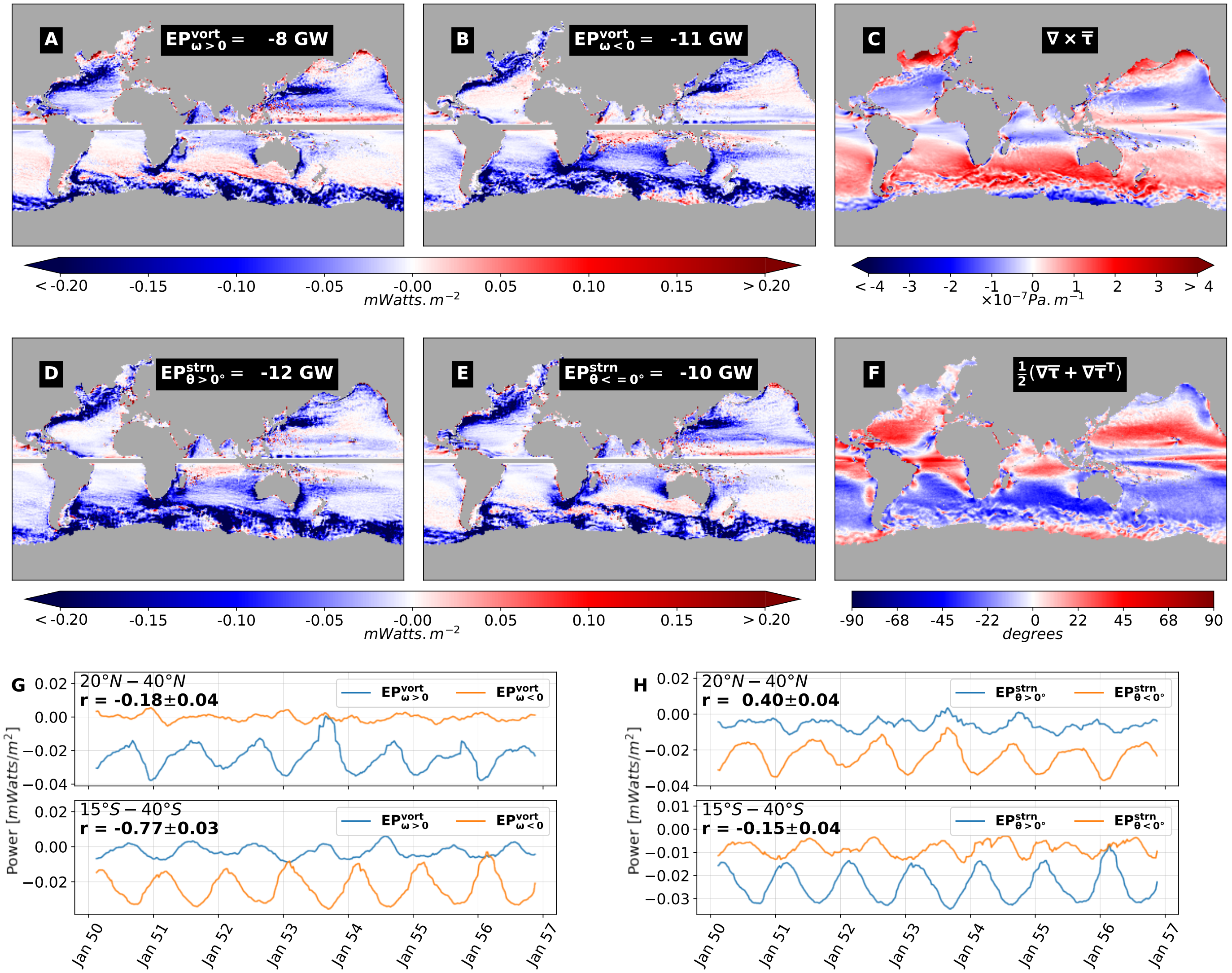

Fig. S8: CESM dataset: Showing the Okubo-Weiss is poor at detecting the inherent asymmetry of energy transfer. Similar to Fig S7, but using Okubo-Weiss to partition the flow into [A-B] vorticity and [D-E] strain regions using a mask function. After calculating EP_ℓ from eq. (1) (in the main text), the mask projects EP_ℓ onto regions of [A] positive vorticity, [B] negative vorticity, [C] positive strain, and [D] negative strain regions. As in Fig S7, positive/negative polarity of strain is based on the angle θ of the local strain's diverging arm (Fig. 2F in the main text). Compared to the corresponding panels in Fig. S7, the panels here are washed out due to the binary nature of the Okubo-Weiss criterion described above in Figs. S2,S9,S4. This is clear from the energization time-series in [G], where negative vorticity in the NH (orange) and positive vorticity in the SH (blue) oscillate around zero in contrast to the corresponding plots in Fig. S7, which are clearly positive and indicate energization of anticyclonic flow by winds in the subtropics. Also missing from [G-H] here is the regular seasonal signal we saw clearly in Fig. S7 along with a significantly weaker correlation coefficients r, which is because a vorticity-dominated region as detected by Okubo-Weiss can also have significant contributions from strain of either sign and vice versa, which contaminate the time-series. Panels [C],[F] of wind stress gradients are the same as in Fig. S7.

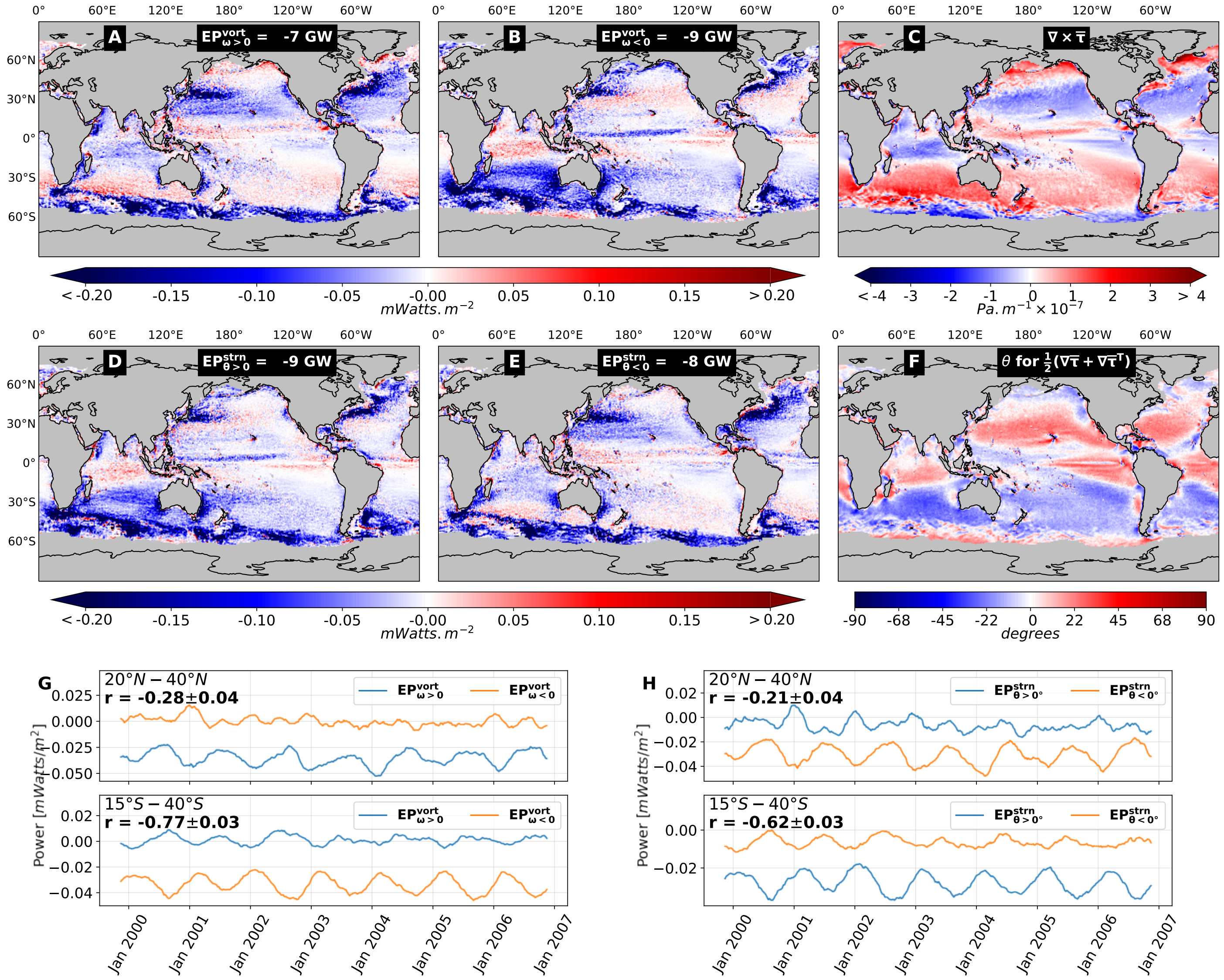

Fig. S9: Okubo-Weiss is poor at detecting the inherent asymmetry of energy transfer. Similar to Fig 3 in the main text, but using Okubo-Weiss to partition the flow into [A-B] vorticity and [D-E] strain regions using a mask function. After calculating EP_ℓ from eq. (1) in the main text, the mask projects EP_ℓ onto regions of [A] positive vorticity, [B] negative vorticity, [C] positive strain, and [D] negative strain regions. As in Fig 3 in the main text, positive/negative polarity of strain is based on the angle θ of the local strain's diverging arm (Fig. 2F in the main text). Compared to the corresponding panels in Fig. 3 in the main text, the panels here are washed out due to the binary nature of the Okubo-Weiss criterion described in the previous Fig. S2. This is clear from the energization time-series in [G], where negative vorticity in the NH (orange) and positive vorticity in the SH (blue) oscillate around zero in contrast to the corresponding plots in Fig. 3 in the main text, which are clearly positive and indicate energization of anticyclonic flow by winds in the subtropics. Also missing from [G-H] here is the regular seasonal signal we saw clearly in Fig. 3 in the main text along with a significantly weaker correlation coefficients r, which is because a vorticity-dominated region as detected by Okubo-Weiss can also have significant contributions from strain of either sign and vice versa, which contaminate the time-series. Panels [C],[F] of wind stress gradients are the same as in Fig. 3 in the main text.

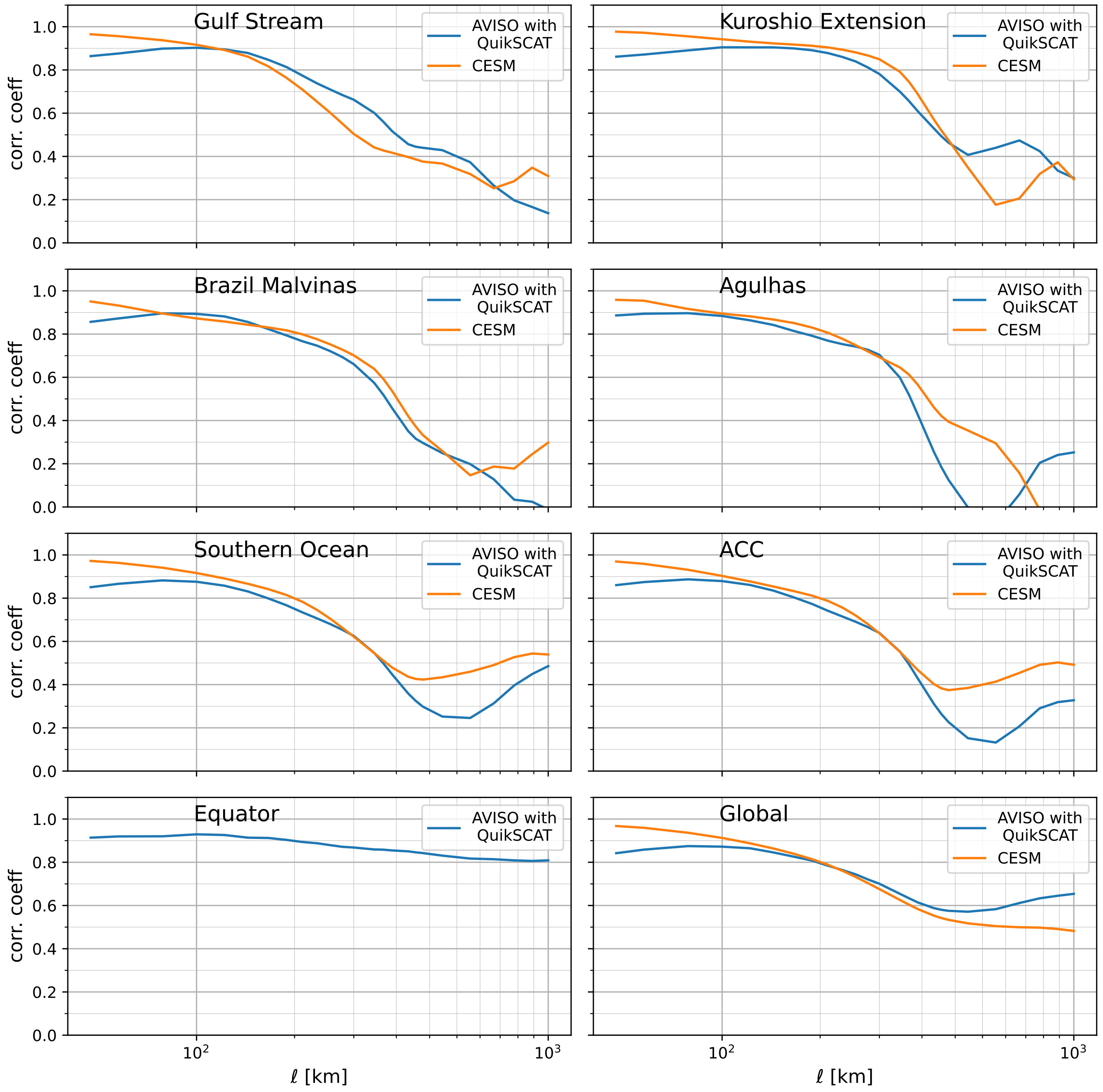

Fig. S10: $\widetilde{EP}_\ell$ is an accurate proxy for EP_ℓ at scales ℓ smaller than ≈ 200 km. As discussed in the Main text and in the Methods, the extent to which $\widetilde{EP}_\ell$ is an accurate proxy for EP_ℓ depends on the latter's ultraviolet locality. Ultraviolet locality of EP_ℓ does not hold at scales around the mesoscale spectral peak ($\ell \approx 300$ km) and larger (see [6, 7]). Panels here plot the correlation between EP_ℓ and $\widetilde{EP}_\ell$, which show that indeed $\widetilde{EP}_\ell$ becomes a poor approximation at those larger scales. An exception is the Equator, where there is no mesoscale spectral peak (see Fig. 1 in [7]). Blue/orange plots show correlation using the satellite/model datasets. Correlation coefficients shown here use single time snapshots shown in Fig 1 [C][D] in the main text and S1[C][D]. The Equator does not show CESM data since we don't calculate the geostrophic ocean velocity from CESM within the band $\pm 3^\circ$.

References for Supplementary Information

[1] Weiss, J. The dynamics of enstrophy transfer in two-dimensional hydrodynamics. *Physica D: Nonlinear Phenomena* **48**, 273–294 (1991).

[2] Okubo, A. Horizontal dispersion of floatable particles in the vicinity of velocity singularities such as convergences. In *Deep sea research and oceanographic abstracts*, vol. 17, 445–454 (Elsevier, 1970).

[3] Small, R. J. *et al.* A new synoptic scale resolving global climate simulation using the community earth system model. *Journal of Advances in Modeling Earth Systems* **6**, 1065–1094 (2014).

[4] Rai, S., Hecht, M., Maltrud, M. & Aluie, H. Scale of oceanic eddy killing by wind from global satellite observations. *Science Advances* **7**, eabf4920 (2021).

[5] Rai, S., Hecht, M. W., Maltrud, M. E. & Aluie, H. Scale-dependent air-sea mechanical coupling: Resolution mismatch and spurious eddy-killing. *ESS Open Archive* (2023).

[6] Storer, B. A., Buzzicotti, M., Khatri, H., Griffies, S. M. & Aluie, H. Global energy spectrum of the general oceanic circulation. *Nature communications* **13**, 5314 (2022).

[7] Storer, B. A., Buzzicotti, M., Khatri, H., Griffies, S. M. & Aluie, H. Global cascade of kinetic energy in the ocean and the atmospheric imprint. *Science Advances* **9**, eadi7420 (2023).
